# Successful thrombolysis with recombinant tissue plasminogen activator after antagonizing dabigatran by idarucizumab: a case report

**DOI:** 10.1186/s13256-016-1050-0

**Published:** 2016-09-29

**Authors:** Annemarie Gawehn, Yassine Ayari, Christian Heuschkel, Matthias Kaste, Pawel Kermer

**Affiliations:** 1 Department of Neurology, Nordwest-Krankenhaus Sanderbusch GmbH, Am Gut Sanderbusch 1, 26452 Sande, Germany; 2 Academic Clinical Department, University Medical Center Gottingen, Gottingen, Germany

**Keywords:** Ischemic stroke, Thrombolysis, Dabigatran, Idarucizumab, recombinant tissue plasminogen activator

## Abstract

**Background:**

Effective anticoagulation routinely precludes patients from receiving intravenous thrombolysis with recombinant tissue plasminogen activator to reverse severe symptoms of ischemic stroke. We report what we believe to be the first case of ischemic stroke successfully treated with recombinant tissue plasminogen activator after antagonizing dabigatran with the monoclonal antibody idarucizumab, recently approved worldwide.

**Case presentation:**

A 75-year-old Caucasian man presented to our hospital with severe aphasia and mild hemiparesis. After providing written consent, he received two doses of 2.5 g of idarucizumab over 20 minutes followed by standard protocol in-label recombinant tissue plasminogen activator application. All symptoms resolved within 1 h.

**Conclusions:**

Applying a recombinant tissue plasminogen activator after antagonizing dabigatran with idarucizumab is feasible and easy to manage in an emergency room or stroke unit. Thus, idarucizumab represents a new therapeutic option for patients receiving dabigatran treatment, reestablishing their eligibility for recombinant tissue plasminogen activator thrombolysis.

## Background

Neurologists have used dabigatran mostly for secondary prevention of cardioembolic stroke in patients with atrial fibrillation since the phase III RE-LY trial [1] showed positive results for the prevention of stroke or systemic embolism as compared with warfarin. Besides dabigatran, three additional non-vitamin-K-dependent oral anticoagulants (NOACs) [2–4] are currently available. Substantially decreased rates of bleeding complications in comparison with warfarin, especially when considering intracerebral hemorrhage, are an advantage of NOAC therapy. However, one major concern was the lack of specific antidotes. With the approval of idarucizumab, a novel monoclonal antibody fragment with a high potency to reverse the anticoagulant effects of dabigatran within minutes [5, 6], the question arose if recombinant tissue plasminogen activator (rt-PA) could be used after reversal of dabigatran with idarucizumab in individuals with newly occurring ischemic stroke. Since the introduction of NOAC therapy, these patients have frequently been excluded from thrombolytic therapy, owing to the unpredictable risk of bleeding.

## Case presentation

A 75-year-old, right-handed Caucasian man with a history of embolic stroke and atrial fibrillation was admitted on 30 January 2016 to the stroke unit of our primary care hospital for severe aphasia and right-sided hemiparesis with sudden onset of symptoms about 1 h before presentation. He was receiving NOAC therapy with dabigatran 110 mg twice daily. His CHA_2_DS_2_-VASc score was 6 points (on scale representing congestive heart failure [or left ventricular systolic dysfunction] 1 point, hypertension [blood pressure consistently above 140/90 mmHg or treated hypertension on medication] 1 point, age ≥75 years 2 points, diabetes mellitus 1 point, prior stroke or transient ischemic attack or thromboembolism 2 points, vascular disease [e.g., peripheral artery disease, myocardial infarction, aortic plaque] 1 point, age 65–74 years 1 point, sex category [female sex] 1 point), and his HAS-BLED score was 3 points (with scale representing hypertension, abnormal renal and liver function, stroke, bleeding, labile international normalized ratio, elderly, drugs or alcohol). Correct dabigatran therapy of 110 mg twice daily could be verified by the accompanying spouse with last intake on the same morning approximately 9.5 h before. Besides cardioembolic stroke, the patient’s medical history consisted of structural epilepsy based on middle cerebral artery ischemia in March 2015 treated with levetiracetam 500 mg twice daily and arterial hypertension treated with amlodipine, metoprolol, and torasemide.

The patient’s impairment of production and comprehension of speech was severe. It was possible for him to communicate, but only to a very limited extent. His hemiparesis was rather mild. His initial National Institutes of Health Stroke Scale (NIHSS) score was 5. No convulsions or lack of consciousness was reported or noticed. According to the patient’s spouse, his symptoms were not comparable to prior seizure symptomatology displayed by sudden loss of consciousness and generalized tonic-clonic convulsions. As a residual of his prior stroke, the patient had a slight outer rotation of his right foot without gait impairment.

**Fig. 1:**
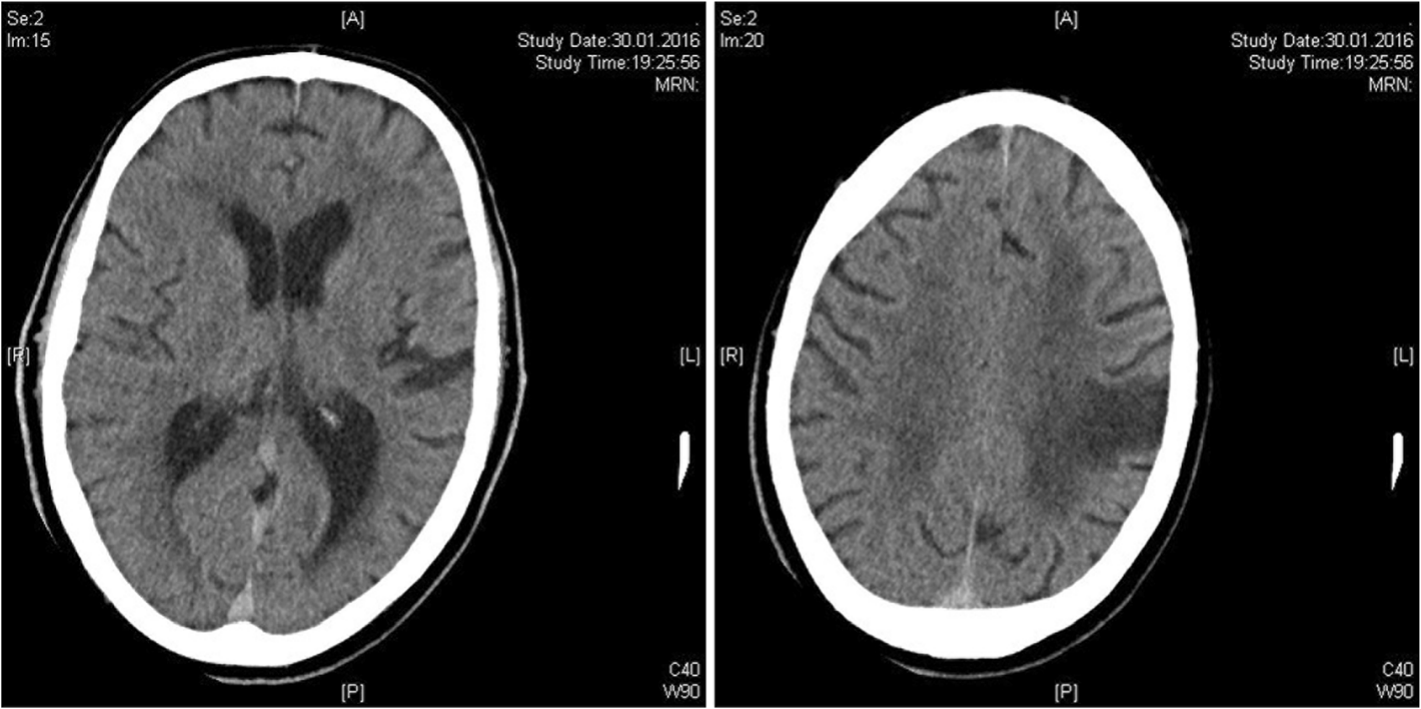
Computed tomographic scan taken upon admission shows no early signs of acute stroke, but displays the residuals of a cardioembolic stroke in the left hemisphere that had occurred in May 2015

Laboratory examinations done upon admission revealed a slight coagulation disorder (international normalized ratio 1.01, activated partial thromboplastin time [aPTT] 39.0 seconds [upper limit of normal 42 seconds], thrombin time 66.8 seconds [upper limit of normal 22 seconds]). Besides a defect zone arising from ischemic stroke in May 2015, computed tomographic (CT) scans (Fig. 1) displayed no early signs of acute stroke. Taking into account the patient’s NIHSS score of 5, no CT angiography or perfusion protocol was performed, in accordance with effective stroke guidelines. Except for the existing oral dabigatran anticoagulation therapy, the patient’s history did not reveal any contraindications against systemic thrombolytic therapy. After the patient provided written consent, we applied two doses of 2.5 g idarucizumab according to the current product characteristics and prescribing information within 20 minutes to antagonize dabigatran. Another blood sample showing normalized coagulation parameters preceded immediate weight-adapted intravenous rt-PA therapy. Within 1 h, all symptoms resolved to baseline levels.

A clinical examination done the next day revealed fluent speech with hardly noticeable word-finding difficulties in an aphasia assessment. Detailed conversation was possible again, and the patient reported subjective well-being. Because the patient’s symptoms resolved within 1 h and because of the lack of therapeutic consequences, we abstained from follow-up magnetic resonance imaging scans as part of the stroke workup. The patient’s electroencephalogram was free of any epileptic discharges. Echocardiography revealed no evidence of intracardial thrombotic material, and an ultrasound of the brain-supplying arteries did not reveal relevant stenoses. With the patient’s low-density lipoprotein cholesterol values being elevated (160 mg/dl), we initiated therapy with simvastatin 20 mg. Dabigatran therapy was reestablished after 24 h. However, on the basis of the patient’s age and normal renal function, we chose an increased dose of 150 mg twice daily according to the effective prescribing information. During idarucizumab application and throughout the entire hospital stay, no thrombotic or bleeding complications occurred. The patient’s laboratory examination results remained normal, and he was released to home in good health.

## Discussion

Intravenous rt-PA thrombolysis in acute ischemic stroke is considered an emergency intervention allowing an in-label application of idarucizumab. Interestingly, in our patient, thrombin time was the only clearly pathologic coagulation parameter 9.5 h after the last medication intake. aPTT was somehow prolonged but still within normal range. A second laboratory examination done immediately after idarucizumab application showed normal coagulation parameters. This allowed in-label use of rt-PA, which is prohibited when anticoagulation therapy is in effect. However, we did not wait for the results, but rather infused rt-PA in order not to lose time to reperfusion of ischemic brain regions. In cases where the second laboratory examinations still show prolonged thrombin time, we suggest immediate termination of rt-PA infusion. However, according to the available idarucizumab data, dabigatran anticoagulation should be abrogated in more than 98 % of patients [6]. Besides our patient, two similar cases [7, 8] have recently been reported. In both cases, rt-PA could be administered successfully after reversing anticoagulation effects of dabigatran by using the specific antidote idarucizumab. As for our patient, no adverse events such as thromboembolism or bleeding occurred in these two cases. Still, large registries on the use of idarucizumab should be established with special emphasis on unfavorable outcomes such as thrombotic events, bleeding, and mortality to further explore potential concerns regarding safety and efficacy.

## Conclusions

To our knowledge, and according to available supplier information, we report the first case worldwide of ischemic stroke successfully treated with rt-PA after antagonizing dabigatran with idarucizumab. In line with two other recent German cases, in which the procedure appeared to be feasible, easy to manage, and without severe adverse events, this novel therapeutic option should be considered in individuals presenting with ischemic stroke who are receiving dabigatran therapy. However, further evidence concerning safety and efficacy is needed before this therapeutic regimen can be generally recommended.
